# Integrating deep learning with multimodal MRI habitat radiomics: toward personalized prediction of risk stratification and androgen deprivation therapy outcomes in prostate cancer

**DOI:** 10.1186/s13244-026-02205-8

**Published:** 2026-01-26

**Authors:** Yun-Feng Zhang, Chuan Zhou, Jia Wang, Han He, Jie Yang, Wenbo Zhang, Hongde Hu, Qidong Wang, Wanbin He, Chao Wang, Rong Wang, Liming Zhao, Fenghai Zhou

**Affiliations:** 1 The First Clinical Medical College of Lanzhou University, Lanzhou, China; 2 Department of Geriatric General Surgery, Sichuan Provincial People’s Hospital, University of Electronic Science and Technology of China, Chengdu, China; 3 The First Clinical Medical College of Gansu University of Chinese Medicine, Lanzhou, China; 4 Department of Radiology, Gansu Provincial Hospital, Lanzhou, China; 5 Department of Urology, Second People’s Hospital of Gansu Province, Lanzhou, China; 6 Department of Urology, Gansu Provincial Hospital, Lanzhou, China

**Keywords:** Prostate cancer risk stratification, Androgen deprivation therapy, Habitat radiomics, Deep learning, Therapeutic response assessment

## Abstract

**Objectives:**

Androgen deprivation therapy (ADT) is essential for treating prostate cancer (PCa) but is limited by tumor heterogeneity. This study develops a non-invasive multiparametric Magnetic Resonance Imaging (mpMRI) radiomics framework to predict ADT response and improve risk stratification.

**Materials and methods:**

A cohort of 550 ADT-treated PCa patients from three centers was analyzed. Patients were randomly divided into training (*n* = 270) and internal validation (*n* = 115) cohorts. An external test cohort (*n* = 165) from Centers 2 and 3 was used for generalizability. Radiomics models based on T2-weighted and diffusion-weighted imaging (DWI), habitat radiomics, and a 3D Vision Transformer (ViT) deep learning model were developed. Ensemble integration of these models was performed, with SHapley Additive exPlanations (SHAP) used for interpretability. Predictive performance was evaluated using receiver operating characteristic (ROC) curves and area under the curve (AUC).

**Results:**

Habitat radiomics outperformed conventional radiomics in Gleason score stratification. For predicting ADT treatment efficacy, the radiomics model achieved AUCs of 0.969 (training), 0.767 (internal validation), and 0.771 (test). The habitat model showed AUCs of 0.987, 0.849, and 0.820, while the ViT model achieved AUCs of 0.831, 0.805, and 0.796. The ensemble model reached the highest AUC of 0.886. SHAP analysis shows that the ViT model contributes most to the combined model, followed by the habitat model, with the radiomics model contributing the least.

**Conclusion:**

mpMRI-based habitat radiomics enables precise risk stratification in PCa. Integrated with conventional radiomics and deep learning, it forms a robust framework for predicting ADT response and guiding personalized treatment.

**Critical relevance statement:**

This study demonstrates that integrating habitat radiomics with deep learning improves the prediction of androgen deprivation therapy response in PCa, advancing personalized radiological decision-making through interpretable multi-model analysis of tumor microenvironment heterogeneity.

**Key Points:**

- Multi-model integration of habitat radiomics and 3D Vision Transformer achieves superior prediction for ADT response compared to conventional methods.
- Habitat radiomics outperforms traditional radiomics in Gleason score stratification.
- SHAP analysis provides clinical interpretability, identifying key model linked to ADT outcomes for actionable insights.

**Graphical Abstract:**

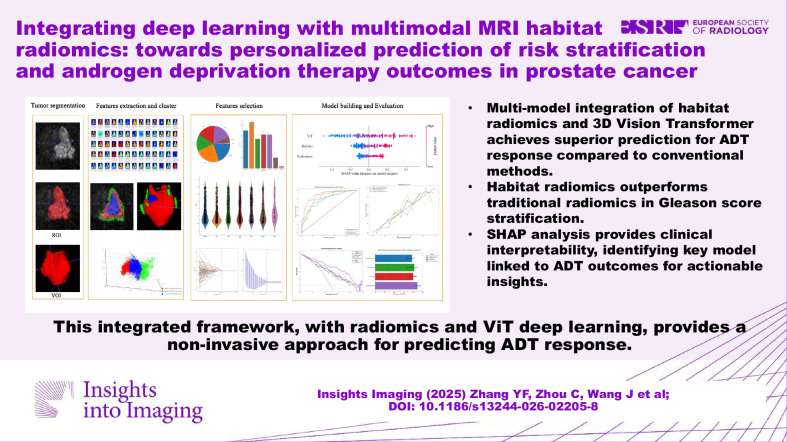

## Introduction

Prostate cancer (PCa), the most common malignancy of the male genitourinary system, is the second most prevalent cancer among males globally [1]. While prostate-specific antigen (PSA) screening is crucial for early detection, its limited sensitivity and specificity often prevent reliable differentiation between benign conditions and clinically significant cancers [2]. As a result, definitive diagnosis relies on invasive prostate biopsy, associated with risks like rectal/perineal hemorrhage, bacteremia, pain, and undersampling of significant lesions [3].

PCa, a hormone-dependent malignancy, is closely linked to androgen levels. Androgen deprivation therapy (ADT) is the cornerstone for treating locally advanced and metastatic PCa, but resistance develops in some patients, with approximately 50% progressing to castration-resistant prostate cancer (CRPC) within 1–3 years [4, 5]. This heterogeneity stems from genomic variations and spatial differences within the tumor microenvironment, such as in vascularization, phenotypic plasticity, and metabolic reprogramming [6]. Therefore, accurately predicting ADT efficacy requires detailed tumor heterogeneity characterization.

Comprehensive tumor heterogeneity characterization demands multi-omics analyses, such as genomics, transcriptomics, and proteomics, but these methods are labor-intensive, costly, and increase patient discomfort due to repetitive sampling [7, 8]. Thus, non-invasive methods with high spatiotemporal resolution are crucial for optimizing PCa management.

Multimodal imaging data integration has facilitated the development of non-invasive digital biopsy, which uses imaging-derived features to predict diagnosis and prognosis [9, 10]. Radiomics, extracting quantitative imaging features, enables non-invasive tumor heterogeneity assessment. However, conventional radiomics often treats tumors as homogeneous, overlooking the distinctiveness of intratumoral subregions, which limits its ability to capture spatial heterogeneity for outcome prediction.

Habitat radiomics overcomes this by using advanced imaging and clustering algorithms to partition tumors into functional subregions (“habitats”), providing spatially resolved profiling for risk stratification and treatment prediction [11]. For example, Chen et al demonstrated MRI-based habitat radiomics for distinguishing breast cancer subtypes based on intratumoral heterogeneity [12].

Vision Transformer (ViT), a deep learning architecture, models global relationships by decomposing images into sequential patches. Unlike traditional CNNs, which focus on local features, ViT captures long-range dependencies, ideal for characterizing spatially distributed tumor heterogeneity [13]. Zhang et al demonstrated ViT’s advantage in predicting treatment responses in esophageal cancer compared to traditional radiomics models [14]. By integrating traditional radiomics, habitat analysis, and ViT deep learning, this approach aims to overcome existing model limitations and capture dynamic changes in the tumor microenvironment, thereby improving the accuracy of ADT outcome prediction. This study introduces a non-invasive framework integrating traditional radiomics, habitat analysis, and 3D ViT using multiparametric MRI to decode tumor heterogeneity, enhancing MRI’s clinical utility for PCa diagnosis and therapeutic stratification.

## Materials and methods

### Study population

**Fig. 1:**
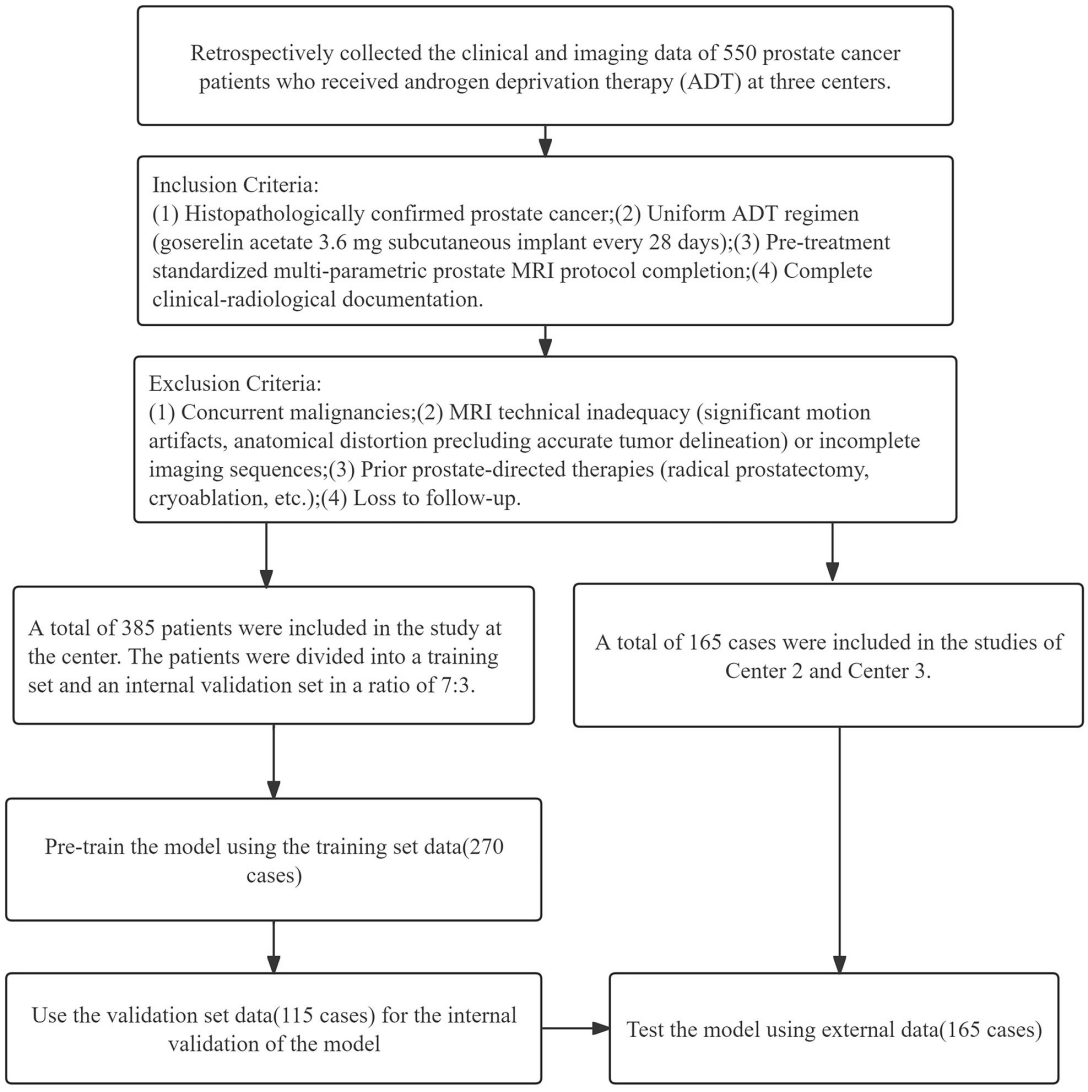
The patient recruitment process of this study

This multi-institutional retrospective study enrolled 385 treatment-naive PCa patients (median age [interquartile range], 73 years [67–78 years]) who underwent ADT at Center 1 between January 2018 and March 2023, with complete pre-therapeutic multiparametric MRI scans and clinicopathological records. All participants received standardized ADT regimens following European Association of Urology (EAU) guidelines. Patients were randomly allocated into training (*n* = 270) and internal validation (*n* = 115) cohorts at a 7:3 ratio. Additionally, an independent external test cohort comprising 165 patients treated at Centers 2 and 3 (January 2021–March 2023) was included for generalizability assessment. The study protocol, compliant with the Declaration of Helsinki, received ethical approval from Center 1 Institutional Review Board (No. 2023-355), with waived informed consent requirements due to anonymized retrospective data analysis. The patient enrollment process is shown in Fig. 1.

Inclusion criteria: (1) Histopathologically confirmed PCa;(2) Uniform ADT regimen (goserelin acetate 3.6 mg subcutaneous implant every 28 days, treatment continued for ≥ 6 cycles unless disease progression (PSA increase > 25% from nadir) or grade ≥ 3 toxicity occurred. To prevent testosterone flare, bicalutamide (50 mg orally daily) was co-administered during the first 28-day cycle.);(3) Pre-treatment standardized multiparametric prostate MRI protocol completion;(4) Complete clinical-radiological documentation.

Exclusion criteria: (1) Concurrent malignancies; (2) MRI technical inadequacy (significant motion artifacts, anatomical distortion precluding accurate tumor delineation) or incomplete imaging sequences; (3) Prior prostate-directed therapies (radical prostatectomy, cryoablation, etc.); (4) Loss to follow-up.

Risk stratification: Following the International Society of Urological Pathology (ISUP) Consensus [15]. PCa cases were stratified into five prognostic tiers: Grade Group 1 (Low-risk): Gleason score (GS) 3 + 3 = 6; Grade Group 2 (Intermediate-low): GS 3 + 4 = 7; Grade Group 3 (Intermediate-high): GS 4 + 3 = 7; Grade Group 4 (High-risk): GS 8; Grade Group 5 (Very high-risk): GS 9–10.

Following ADT, 264 patients exhibited significant tumor regression, manifested by: 1. It was observed that the symptoms related to the tumor (such as difficulty in urination and bone pain) have improved, based on the numerical rating scale (NRS).2. The imaging examination results showed that the tumor volume has significantly decreased, and the lymph node metastasis foci and bone metastasis foci have significantly reduced.3. After the treatment, the level of PSA decreased by 50% or more (ideally to below 0.2 ng/mL).

### Prostate tumor segmentation

The image acquisition parameters and preprocessing methods are provided in Supplementary Material 1. Following image preprocessing, precise segmentation of PCa lesions was performed on two core sequences—T2WI and DWI—using multimodal image fusion. Three-dimensional (3D) interactive annotation was conducted by two radiologists (each with over 10 years of experience in genitourinary imaging) using the ITK-SNAP platform (v4.0.0). Anatomical constraints were applied: the prostate capsule on T2WI served as the boundary, and contour tracking was performed along the maximal gradient direction at the tumor-normal tissue interface. Critical structures, including the urethral sphincter complex (with a 2 mm safety margin from the mucosa), verumontanum, and seminal vesicle transitional zones, were avoided. For multifocal tumors, the index lesion (largest focus ≥ 15 mm) was prioritized per clinical guidelines, with spatial consistency across sequences ensured through 3D registration. The software automatically fused multi-layer ROIs to generate 3D volumetric regions of interest (VOIs), exporting mask files in nii.gz format for analysis.

To assess the reproducibility of manual segmentation, a validation set of 35 samples was established. Two experienced radiologists (each with over 10 years of expertise in prostate MRI diagnosis) independently delineated tumor regions of interest (ROIs) while blinded to pathological outcomes. Spatial concordance was evaluated using the Dice similarity coefficient, Jaccard index, Hausdorff distance (95th percentile), and Cohen’s Kappa for non-random agreement. The mean Dice coefficient was 0.86 ± 0.04, the median Jaccard index was 0.79, the 95th percentile Hausdorff distance was 1.87 mm, and Cohen’s Kappa was 0.83, confirming strong consistency of the segmentation protocol. All subsequent analyses used consensus segmentation results, with discordant cases resolved through mutual discussion.

### Habitat generation and feature extraction

After completing the ROI delineation, radiomic features were extracted from both T2-weighted imaging (T2WI) and diffusion-weighted imaging (DWI) sequences using PyRadiomics. Adaptive clustering to generate habitat regions: To capture intratumoral heterogeneity, spatially continuous habitat regions were generated through an optimized k-means clustering algorithm. The specific algorithm can be found in Supplementary Material 2.

### Feature selection and signature construction

#### Radiomics signature

Z-score normalization is applied to standardize the dataset (column = (column − mean) / std). Pearson’s correlation coefficient identifies strong correlations, retaining only one feature when the correlation exceeds 0.9. A reliable feature set is established for further analysis. Stepwise search based on the LASSO algorithm selects the optimal feature combination based on accuracy, with multiple iterations evaluating feature importance. Prediction models are constructed using various machine learning classifiers.

### Deep learning signature

**Fig. 2:**
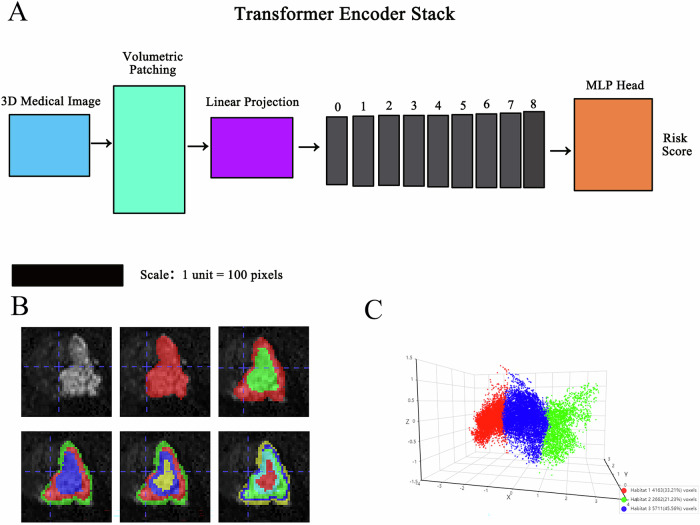
**A** Schematic diagram of ViT model training. **B** Generation of habitat subregions. **C** Three-dimensional visualization of habitat features

Volumetric regions of interest (VOIs) encompassing tumor lesions were extracted from the original images using a validated 3D cropping tool, which were saved in the standardized nii.gz format for subsequent deep learning model pre-training. As illustrated in Fig. 2A, the specific parameters can be found in Supplementary Material 3.

### Model selection

Model selection focused on test-set performance, complemented by statistical significance testing and generalization evaluation. Candidate models with superior performance in core metrics—such as AUC, accuracy, F1 score, PPV, and NPV—on the test set were initially selected. DeLong tests assessed the statistical significance of AUC differences, while overfitting risks were evaluated, prioritizing models with minimal performance disparity between the training and test sets. The optimal model was chosen based on a balanced evaluation of sensitivity, specificity, and weighted metric proportions.

Notably, Within the model evaluation framework, five core dimensions and their criteria were defined: Discriminative performance required an AUC exceeding 0.70, weighted at 40%; calibration was assessed by the Brier Score with a threshold below 0.05, weighted at 15%; generalization capability was evaluated by the AUC reduction between the validation and training sets (less than 0.05), contributing 20%; clinical utility was determined by DCA at a 95% confidence interval, with a DCA value above 0.3, weighted at 15%; and interpretability was measured by the Dice coefficient of the ROI, requiring a coefficient above 0.6, representing 10%.

### Model integration

The prediction probabilities generated by individual models for each sample were standardized using either Z-score normalization or Min-Max normalization to eliminate scale discrepancies. Given that Logistic Regression (LR) inherently produces probability outputs—facilitating subsequent integration with clinical risk scoring systems—we utilized an LR classifier to train a unified ensemble model. In this framework, true clinical labels served as the dependent variable, while the standardized probabilities derived from three base models functioned as independent predictors. To mechanistically interpret the ensemble model, we applied SHapley Additive exPlanations (SHAP) analysis to quantify feature contributions and visualize decision pathways.

### Statistical analysis

Statistical analyses were performed using SPSS 23.0 and R software version 3.6.1. Normality and homogeneity of variance for continuous variables were assessed via the Kolmogorov–Smirnov test. Normally distributed data with equal variances were expressed as mean ± standard deviation (SD) and compared between groups using independent samples *t*-tests. For non-normally distributed data or unequal variances, non-parametric Mann–Whitney U tests were applied for intergroup comparisons. A two-tailed *p*-value < 0.05 was considered statistically significant.

## Results

### Patient cohort and clinical characteristics

A total of 550 patients were enrolled in the cohort study, stratified by GS groups: GS 6 (*n* = 38), GS 3 + 4 (*n* = 58), GS 4 + 3 (*n* = 46), GS 8 (*n* = 168), and GS 9–10 (*n* = 230). Statistical analysis confirmed no significant differences in baseline clinical characteristics between the training, internal validation, and external test cohorts (Table 1).

**Table 1:** Clinical information of patients in the training set, internal validation set, and external test set

Variables	Total (*n* = 550)	External test (*n* = 165)	Internal dataset (*n* = 385)					Statistic	*p*-value
			Total (*n* = 385)	Train (*n* = 270)	Val (*n* = 115)	Statistic	*p*-value		
Outcome, *n* (%)						χ² = 0.00	0.954	χ² = 0.00	0.970
Ineffective treatment	286 (52.00)	86 (52.12)	200 (51.95)	140 (51.85)	60 (52.17)				
Effective treatment	264 (48.00)	79 (47.88)	185 (48.05)	130 (48.15)	55 (47.83)				
GS, *n* (%)						χ² = 2.92	0.571	χ² = 1.38	0.847
6	38 (6.91)	14 (8.48)	24 (6.23)	19 (7.04)	5 (4.35)				
3 + 4 = 7	58 (10.55)	17 (10.30)	41 (10.65)	28 (10.37)	13 (11.30)				
4 + 3 = 7	56 (10.18)	15 (9.09)	41 (10.65)	32 (11.85)	9 (7.83)				
8	168 (30.55)	48 (29.09)	120 (31.17)	80 (29.63)	40 (34.78)				
9–10	230 (41.82)	71 (43.03)	159 (41.30)	111 (41.11)	48 (41.74)				
Age (years)	73.00 (67.00, 78.00)	73.00 (67.00, 78.00)	73.00 (67.00, 78.00)	73.00 (67.00, 77.75)	74.00 (66.50, 79.00)	Z = −1.14	0.252	Z = −0.35	0.729
Tpsa (ng/mL)	57.36 (19.86, 100.00)	48.84 (17.42, 100.00)	61.34 (21.14, 100.00)	61.34 (20.77, 100.00)	61.42 (21.69, 100.00)	Z = −0.01	0.993	Z = −1.53	0.125
FPSA (ng/mL)	8.23 (2.48, 29.00)	7.27 (1.85, 27.62)	8.37 (2.85, 29.40)	8.37 (2.80, 27.79)	8.22 (3.03, 30.00)	Z = −0.00	0.996	Z = −1.35	0.175
Volume (mL)	41.00 (31.35, 61.75)	42.40 (30.00, 67.00)	40.60 (31.90, 61.00)	41.00 (31.92, 61.75)	39.00 (31.65, 60.00)	Z = −0.44	0.659	Z = −0.51	0.611
PSAD (ng/mL^2^)	1.14 (0.46, 2.25)	1.02 (0.38, 1.92)	1.25 (0.52, 2.33)	1.25 (0.54, 2.21)	1.25 (0.43, 2.48)	Z = −0.40	0.689	Z = −1.83	0.067
BMI (kg/m^2^)	23.20 (21.20, 25.34)	23.39 (21.22, 25.71)	23.16 (21.16, 25.26)	23.14 (21.19, 25.29)	23.26 (21.15, 25.26)	Z = −0.33	0.739	Z = −1.06	0.289
TP (g/L)	68.25 (63.51, 72.60)	68.30 (64.20, 72.50)	68.08 (63.20, 72.60)	68.55 (63.27, 72.66)	67.60 (63.05, 71.45)	Z = −1.09	0.275	Z = −0.77	0.444
ALB (g/L)	40.00 (37.13, 43.30)	40.27 (37.20, 43.44)	39.90 (36.98, 43.30)	39.95 (37.03, 42.90)	39.90 (36.45, 43.47)	Z = −0.07	0.947	Z = −0.77	0.444
ALP (U/L)	86.00 (68.21, 130.00)	82.86 (66.24, 130.00)	87.00 (69.89, 130.00)	88.50 (71.07, 131.75)	84.33 (65.00, 118.00)	Z = −1.44	0.149	Z = −0.96	0.337
UA (mg/mL)	320.00 (267.35, 375.32)	325.00 (267.00, 375.35)	318.59 (268.38, 375.00)	311.00 (269.00, 376.00)	337.00 (265.21, 372.00)	Z = −0.49	0.626	Z = −0.23	0.821
Ca (mmol/L)	2.22 (2.15, 2.32)	2.22 (2.17, 2.32)	2.22 (2.15, 2.32)	2.22 (2.15, 2.31)	2.24 (2.15, 2.33)	Z = −1.16	0.246	Z = −0.67	0.504
P (mmol/L)	1.06 (0.94, 1.22)	1.06 (0.94, 1.19)	1.07 (0.94, 1.24)	1.07 (0.93, 1.25)	1.06 (0.95, 1.20)	Z = −0.14	0.892	Z = −0.62	0.535
Fbg (g/L)	3.50 (2.89, 4.42)	3.34 (2.90, 4.36)	3.58 (2.87, 4.46)	3.59 (2.84, 4.57)	3.50 (3.10, 4.29)	Z = −0.25	0.806	Z = −1.43	0.154
NEUT (× 10^9^/L)	3.64 (2.78, 4.80)	3.69 (2.84, 4.94)	3.62 (2.77, 4.79)	3.62 (2.81, 4.66)	3.59 (2.74, 5.03)	Z = −0.62	0.536	Z = −0.32	0.746
M (× 10^9^/L)	0.50 (0.38, 0.71)	0.50 (0.39, 0.74)	0.50 (0.38, 0.69)	0.47 (0.38, 0.67)	0.54 (0.38, 0.93)	Z = −1.60	0.109	Z = −0.39	0.694
Lym (× 10^9^/L)	1.21 (0.70, 1.66)	1.23 (0.70, 1.83)	1.21 (0.70, 1.65)	1.23 (0.72, 1.66)	1.07 (0.52, 1.60)	Z = −1.36	0.175	Z = −1.08	0.282
Hb (g/L)	142.00 (126.00, 154.00)	142.00 (129.00, 154.00)	142.00 (124.00, 154.00)	141.00 (122.25, 154.00)	144.00 (126.00, 154.00)	Z = −0.88	0.376	Z = −0.87	0.385
PLT (× 10^9^/L)	182.00 (149.00, 223.00)	184.00 (156.00, 226.00)	179.00 (144.00, 223.00)	180.00 (144.25, 223.00)	179.00 (143.50, 222.50)	Z = −0.27	0.789	Z = −1.50	0.134

*GS* Gleason score, *Tpsa* total prostate-specific antigen, *FPSA* free prostate-specific antigen, *PSAD* PSA density, *BMI* body mass index, *TP* total protein, *ALB* albumin, *ALP* alkaline phosphatase, *UA* uric acid, *Ca* calcium, *P* phosphorus, *Fbg* fibrinogen, *NEUT* neutrophil, *M* monocyte, *Lym* lymphocyte, *Hb* hemoglobin, *PLT* platelet, *Z* Mann–Whitney U test, *χ²* Chi-square test

### ROI cluster and feature selection

**Fig. 3:**
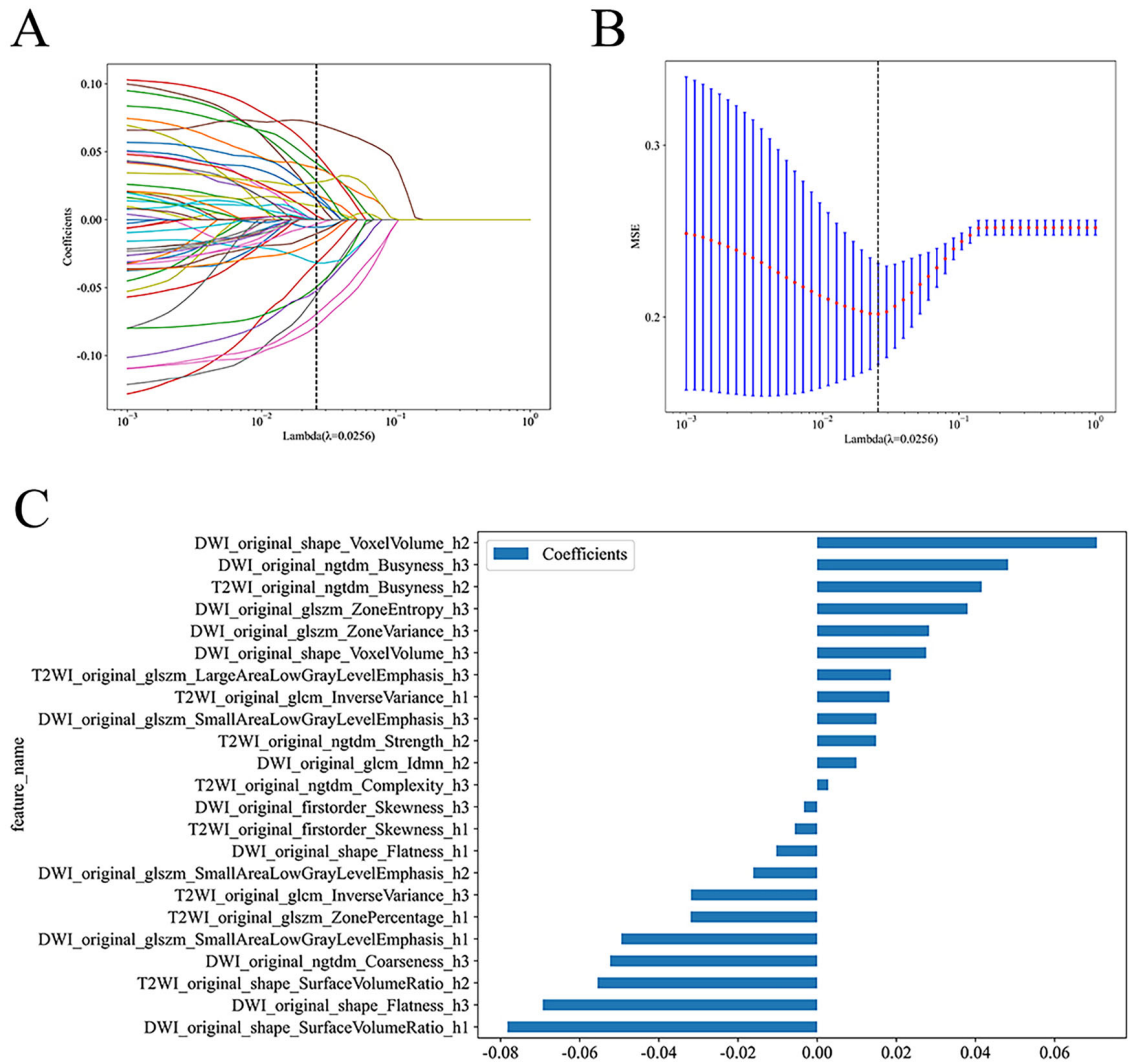
The process of feature selection (taking the process of selecting habitat features for predicting the effect of ADT as an example). **A** Shows the coefficients obtained through Lasso (Least Absolute Shrinkage and Selection Operator) in ten-fold cross-validation, which are applied to habitat radiomics feature analysis. **B** Lasso path of MSE in the ten-fold cross-validation of habitat radiomics features. **C** Final selected features and their corresponding weight coefficients

This study utilized 108 features, including grayscale values, to characterize ROIs based on two sequences. After saving them as npy files, habitat region clustering was performed (2–5 clusters, Fig. 2B), with peak performance at cluster = 3. Habitat features were then visualized in three dimensions (Fig. 2C). Radiomics feature extraction was conducted based on the original ROI and habitat subregions. Feature fusion used the “fill” method, clustering all samples. Hyperparameters, such as the number of features, were determined using 5-fold cross-validation. LASSO regression selected features closely associated with outcome indicators (Fig. 3).

### Model evaluation

**Fig. 4:**
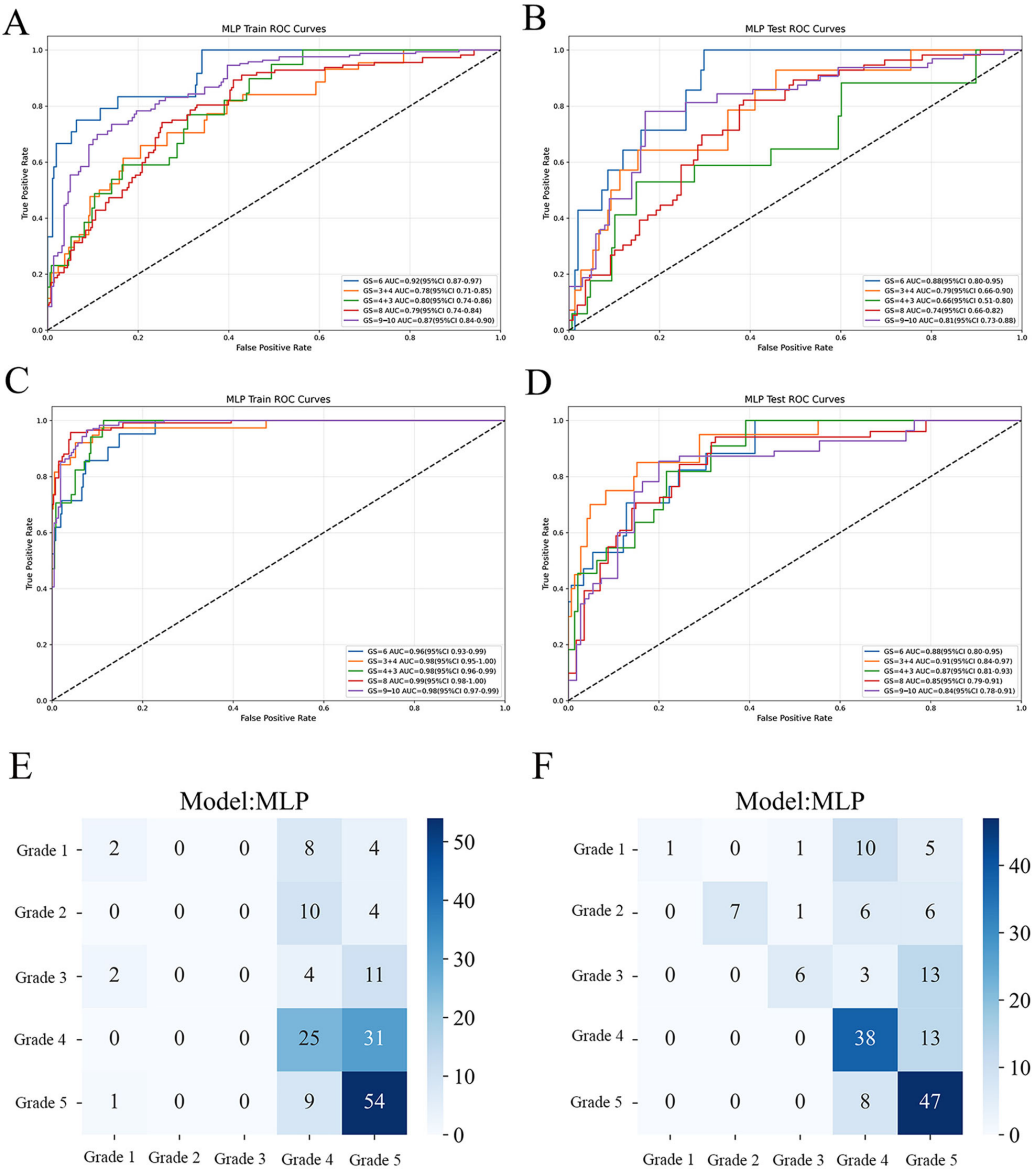
Receiver operating characteristic (ROC) curve analysis for multi-class prediction of GS (Gleason score). **A** Radiomics model training set, **B** Radiomics model testing set, **C** Habitat model training set, **D** Habitat model testing set. Confusion matrix. **E** Radiomics model: low grade (Grade 1–3) is almost entirely misclassified into high grade (Grade 4–5), with a tendency for overestimation, and there is considerable confusion between Grade 4 and 5 (e.g., Grade 4→5 occurred 31 times). **F** Habitat model: improved prediction of mid-grade (Grade 2–3), reducing complete misclassification and significantly reducing misclassification from Grade 4→5 (from 31 times down to 13 times), leading to more accurate classification. However, the prediction for Grade 1 is more conservative (recall rate further reduced)

In this study, machine learning classifiers were employed to construct both conventional radiomics and habitat radiomics models. The multi-layer perceptron classifier demonstrated optimal performance for GS prediction (Fig. 4A–D). For the conventional radiomics model, the test set AUCs were as follows: GS 6: 0.88 (95% CI 0.80–0.95), GS 3 + 4: 0.79 (95% CI 0.66–0.90), GS 4 + 3: 0.66 (95% CI 0.51–0.80), GS 8: 0.74 (95% CI 0.66–0.82), and GS 9–10: 0.81 (95% CI 0.73–0.88). In contrast, the habitat radiomics model achieved superior test set performance: GS 6: 0.88 (95% CI 0.80–0.95), GS 3 + 4: 0.91 (95% CI 0.84–0.97), GS 4 + 3: 0.87 (95% CI 0.81–0.93), GS 8: 0.85 (95% CI 0.79–0.91), and GS 9–10: 0.84 (95% CI 0.78–0.91).

The habitat radiomics model exhibited enhanced predictive capability across all GS categories compared to conventional radiomics, particularly improving precision in intermediate-to-high-grade stratification. Confusion matrix analysis further confirmed its superiority in overall accuracy and high-risk PCa identification (Fig. 4E, F).

**Fig. 5:**
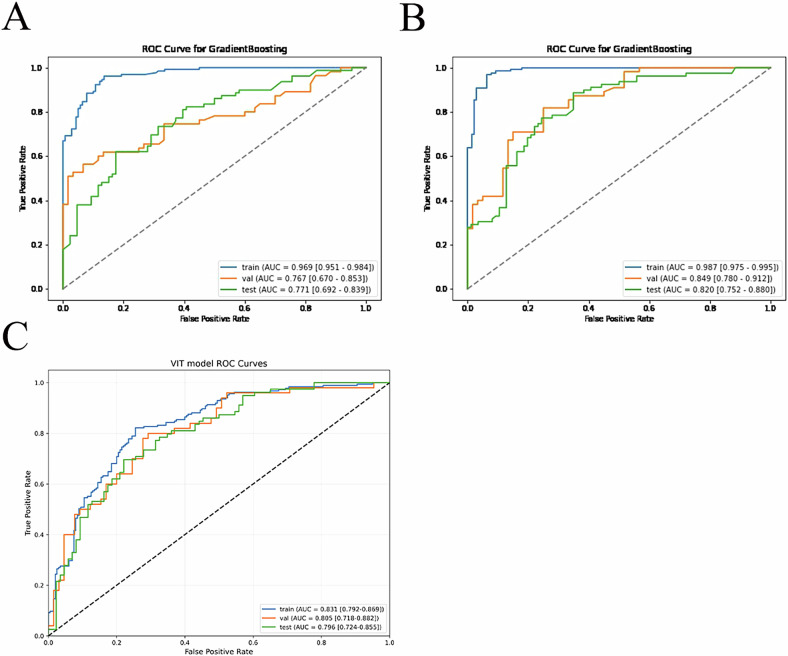
**A** ROC curve analysis of the radiomics model for predicting ADT treatment outcomes; **B** ROC curve analysis of the habitat model for predicting ADT treatment outcomes; **C** ROC curve of the ViT model

Importantly, this study developed a predictive model for ADT treatment efficacy based on radiomics, habitat analysis, and the ViT deep learning model. The specific parameters of different classifier models and the results of the Delong test are presented in Supplementary Tables 1–4. The best machine learning classifier was GradientBoosting, which achieved an AUC of 0.969 (95% CI 0.951–0.984) on the training set of the radiomics model (Fig. 5A), an AUC of 0.767 (95% CI 0.670–0.853) on the internal validation set, and an AUC of 0.771 (95% CI 0.692–0.839) on the external test set. For the habitat analysis model (Fig. 5B), the training set AUC was 0.987 (95% CI 0.975–0.995), the internal validation set AUC was 0.849 (95% CI 0.780–0.912), and the external test set AUC was 0.820 (95% CI 0.752–0.880). The ViT-based 3D deep learning model (Fig. 5C) had an AUC of 0.831 (95% CI 0.791–0.870) on the training set, an AUC of 0.805 (95% CI 0.718–0.882) on the internal validation, and an AUC of 0.796 (95% CI 0.725–0.850) on the external test set. Additionally, during the development of this study, we also used ReaNet and DenseNet series to build deep learning models, but these models suffered from severe overfitting and poor generalization ability.

### Model integration

**Fig. 6:**
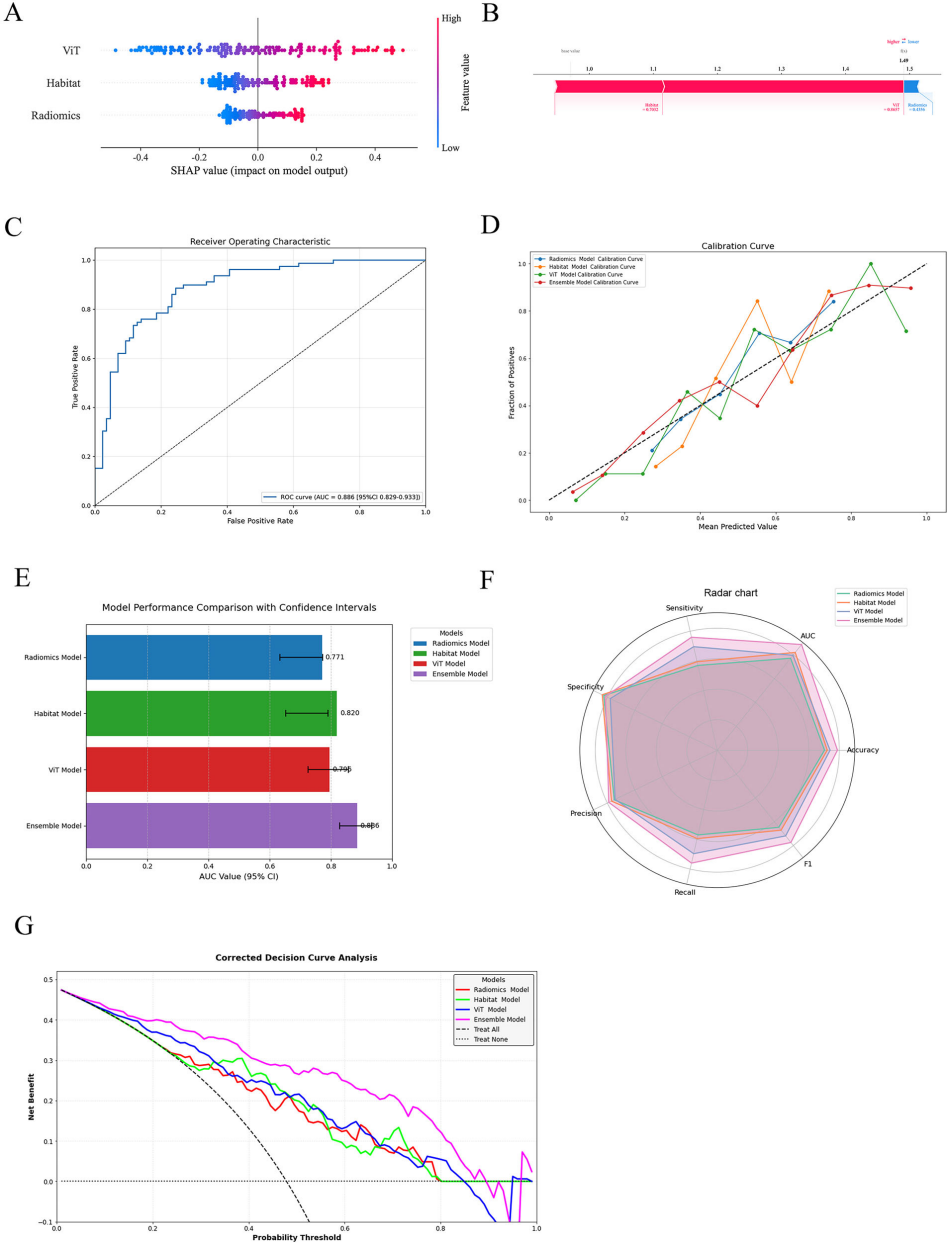
**A** The SHapley Additive exPlanations (SHAP) summary plot of the ensemble model, which elucidates the interplay between constituent models and attributes the predictive performance to their combined feature contributions, reveals that in this study, the VIT model made the largest contribution to the ensemble, followed by the habitat model, with the radiomics model exhibiting the smallest influence; **B** the SHAP force plot explains how the ensemble model distinguishes the treatment response of an individual patient. **C** Ensemble model evaluation ROC curve analysis, **D** the calibration curve indicates that the combined model has a better fit; **E** confidence interval bar chart; **F** the radar chart shows that the combined model has better overall performance; **G** the decision curve analysis (DCA) indicates that the combined model has greater clinical application value

To enhance ADT response prediction accuracy, we integrated predictions from radiomics, habitat radiomics, and 3D ViT models using an LR classifier, constructing a unified ensemble model. SHAP analysis was applied to decode the LR model’s decision logic, generating a global feature importance summary plot. This plot ranks features by their mean absolute SHAP values (descending order), quantifying their aggregate predictive contributions. Individual patient SHAP values are visualized as horizontally distributed points, with vertically stacked density reflecting frequency distribution. Color mapping (low feature values: blue; high values: red) reveals nonlinear relationships between feature magnitudes and their predictive impact, while point dispersion along the horizontal axis indicates directional effects (positive/negative predictive contributions). By translating the LR model’s linear mechanisms into interactive visualizations, this approach bridges statistical rigor with clinical interpretability, enabling biologically plausible decision support. Figure 6A, B highlights the ViT model as the most influential contributor to ensemble predictions, followed by habitat radiomics. The color gradient in ViT-derived SHAP values demonstrates monotonically increasing predictive weight with elevated feature values.

The ensemble model was evaluated using ROC curves, calibration plots, and confidence interval bar charts, achieving a superior AUC of 0.89 (95% CI: 0.829–0.933) (Fig. 6C–E), with DeLong’s test confirming statistically significant AUC improvements (*p* < 0.05). Radar plots further demonstrate its dominance across performance metrics—sensitivity, specificity, precision, recall, accuracy, and F1 score—over single-modality models (Fig. 6F). DCA corroborates enhanced clinical net benefit across threshold probabilities (Fig. 6G), validating its translational utility.

## Discussion

ADT remains a cornerstone in PCa management, particularly for high-risk, locally advanced, or metastatic cases, where it significantly prolongs survival and improves quality of life [16]. However, intrinsic resistance to ADT in some patients requires timely therapeutic adjustments, such as using novel endocrine agents or transitioning to chemoradiotherapy. Inappropriate ADT duration can also lead to long-term morbidity, including bone density loss and increased fracture risk [17]. Therefore, predicting ADT response is crucial for optimizing therapeutic strategies, minimizing complications, and improving patient outcomes.

Divergent responses to pharmacotherapy reflect intrinsic tumor heterogeneity, manifested as genomic, transcriptomic, phenotypic, metabolic, and functional diversity. Even within individual tumors, treatment-resistant subclones may emerge, influencing key oncological behaviors—such as invasiveness, metastasis, recurrence, and therapeutic resistance—that directly affect clinical outcomes [18, 19]. Thus, profiling tumor heterogeneity may enable more precise diagnostic stratification and prognostic evaluation, shifting toward personalized therapies.

Advancements in artificial intelligence and medical imaging have made radiomics a powerful tool for analyzing tumor microarchitecture through high-throughput feature extraction. For instance, Wang et al showed that radiomics could predict prognosis in locally advanced breast cancer by reflecting tumor cell and microenvironmental heterogeneity [20]. Jiang et al demonstrated its utility in evaluating tumor microenvironment and predicting treatment response for gastric cancer [21]. MRI-based radiomics is also widely used in PCa diagnosis and prognostic evaluation [22], though it often overlooks spatial intratumoral heterogeneity.

Habitat radiomics addresses this limitation by partitioning tumors into distinct subregions or “habitats,” each reflecting unique biological properties such as necrosis and hypoxia. This approach views tumors as heterogeneous ecosystems, influencing therapeutic responses and outcomes. By doing so, habitat radiomics enhances predictive accuracy and supports personalized treatment strategies. Wu et al used CT-based habitat radiomics to predict progression-free survival in non-small cell lung cancer [23]. Yang et al showed MRI radiomics with habitat analysis improved machine learning-based predictions of bone metastasis and high-grade GS in PCa [24]. Superior prognostic performance has also been reported for hepatocellular carcinoma, breast cancer, and glioblastoma [25–27]. In this study, tumor habitats were characterized using 108 radiomic features and adaptive clustering (K = 2–5). Peak performance was achieved with K = 3 clusters, which provided superior risk stratification and ADT response prediction compared to conventional radiomics.

The ViT model offers distinct advantages in medical radiomics by capturing global contextual information through self-attention mechanisms, a capability crucial for detecting subtle lesions in medical images. ViT adapts to focus on biologically relevant regions like tumor heterogeneity zones, improving diagnostic precision. For example, Niu et al used ViT to discriminate glioma subtypes [28], while Jiang et al showed ViT-based deep learning outperformed CNNs in predicting survival for rectal cancer [29]. Chaurasia et al further demonstrated that self-supervised ViT models can leverage histological images to effectively diagnose and grade PCa, differentiating between benign and malignant tissues while categorizing tumors by malignancy degree [30]. In this study, ViT was trained on 3D regions, enhancing predictive performance by preserving richer spatial-contextual information.

SHAP was used to explain the ensemble model, providing transparent, reliable interpretability based on game theory. It quantifies the contribution of each feature, ensuring consistency between global model behavior and local predictions. SHAP has been applied to multiple disease prediction models [31, 32]. In this study, we compared the contributions of different models using SHAP for quantitative analysis.

This study has limitations. Despite being a multi-center investigation, the sample size is small with short follow-up, limiting long-term observations. Imbalanced sample distribution across subgroups may also introduce stratification bias. Manual tumor annotation is time-consuming; future work should prioritize automated segmentation models for better efficiency and consistency. Nevertheless, our framework demonstrates strong performance in comprehensive PCa assessment, and with further AI advancements, it holds promise for improving clinical PCa management.

## Conclusion

In conclusion, mpMRI-based habitat radiomics, integrated with conventional radiomics and ViT deep learning, provides a non-invasive framework for predicting ADT response and guiding personalized treatment strategies.

## Supplementary information

ELECTRONIC SUPPLEMENTARY MATERIAL

## Data Availability

Due to patient privacy protection, the clinical and MRI image datasets used in this study will not be made publicly available.

## Abbreviations

ADT: Androgen deprivation therapy
AUC: Area under the curve
DCA: Decision curve analysis
DWI: Diffusion weighted imaging
GS: Gleason score
Lasso: Least Absolute Shrinkage and Selection Operator
M: Monocyte
mpMRI: Multiparametric magnetic resonance imaging
PCa: Prostate cancer
PSA: Prostate-specific antigen
ROC: Receiver operating characteristic
ROI: Regions of interest
TP: Total protein
ViT: Vision Transformer

## Code availability

The code provided in this study was supplied by the Onekey AI platform and can be reasonably requested from the corresponding author.
