# Supplementary material for: Integrating deep learning with multimodal MRI habitat radiomics: toward personalized prediction of risk stratification and androgen deprivation therapy outcomes in prostate cancer
Source: Insights Imaging. 2026 Jan 26;17:16. doi: 10.1186/s13244-026-02205-8 (PMC12834885; doi:10.1186/s13244-026-02205-8)
Supplement: Supplementary file 1 — ELECTRONIC SUPPLEMENTARY MATERIAL [file 13244_2026_2205_MOESM1_ESM.pdf]

Integrating Deep Learning with Multimodal MRI Habitat Radiomics: Towards Personalized Prediction of Risk Stratification and Androgen Deprivation Therapy Outcomes in Prostate Cancer

ELECTRONIC SUPPLEMENTARY MATERIAL

Image Acquisition Protocol

All MRI examinations were performed on a 3.0T superconducting MRI system equipped with a 32-channel phased-array torso coil. The standardized multi-parametric prostate MRI protocol included the following core sequences: Axial T2-weighted Imaging (T2WI):Pulse sequence: Turbo spin-echo (TSE);Acquisition parameters: TR/TE = 2800/120 ms, slice thickness/gap = 3.0/0.6 mm;Spatial resolution: Matrix = 256×256, FOV = 200×200 mm²;Signal optimization: Number of signal averages (NSA) = 2, parallel imaging using sensitivity encoding (SENSE) factor = 2. Axial Diffusion-weighted Imaging (DWI):Pulse sequence: Single-shot echo-planar imaging (EPI);Diffusion encoding: Three orthogonal directions with b-values = 0, 800, 1500 s/mm²;Acquisition parameters: TR/TE = 4000/60 ms, contiguous 3.0 mm slices;Spatial resolution: Matrix = 128×128.

Image Preprocessing Pipeline

Spatial Normalization:Isotropic resampling to 1 mm^3 voxel dimensions using third-order B-spline interpolation (ITK framework) to preserve anatomical continuity.Intensity Harmonization:N4 bias field correction (ANTs toolkit) with 200 iterations and convergence threshold = 0.001 to mitigate magnetic field inhomogeneity effects.Spatial Registration:Rigid-body transformation aligned all sequences to the standard RAS coordinate system based on DICOM header metadata, ensuring submillimeter multi-modal spatial consistency.

Quality control system

Implementation of automated Image Quality Assessment (IQA): Calculation of quantitative indicators such as whole-brain signal-to-noise ratio (SNR ≥ 15), relative uniformity (RU ≥ 85%), etc. Manual review: Two senior radiologists assess anatomical structure visibility using a double-blind method (according to the PI-RADS v2.1 standard), excluding cases with motion artifacts or blurred key anatomical landmarks.

SUPPLEMENTARY MATERIAL 2

The process began with the random selection of K initial cluster centroids from tumor voxels. For each voxel V_{ijk}, the Euclidean distance to all centroids was computed, and the voxel was assigned to the nearest cluster. Cluster centers were then iteratively updated by calculating the mean coordinates of their member voxels until convergence (i.e., no further voxel reassignment) or until reaching a predefined maximum iteration limit (e.g., 100 cycles). The optimal number of habitats (K = 2–5) was determined by evaluating the elbow method to minimize the within-cluster sum of squares, supplemented by silhouette score analysis to validate biological relevance and spatial coherence.

Local and global feature extraction: Local entropy feature calculation: For each voxel Vijk, local entropy is computed within its 3×3×3 neighborhood (26 adjacent voxels) to quantify spatial heterogeneity. Similarly, PyRadiomics is used to extract radiomics features from different subregions of the habitat.

Feature fusion and habitat characterization: The habitat features from different modalities are concatenated to generate a unified radiomics feature vector. For modality *m*, the habitat feature is denoted as $habitat_m$, and the fused feature vector is:

$habitat_{feat}=habitat_{m1}\oplus habitat_{m2}\oplus\cdots\oplus habitat_{mn}$,

Where $\oplus$ represents feature concatenation.

SUPPLEMENTARY MATERIAL 3

a ViT-based pre-training framework was implemented with the following architectural specifications: input image size = 64 × 64 × 48 (height × width × depth), spatial patch size = 16 × 16, temporal patch size = 2 slices, embedding dimension (dim) = 1024, transformer depth = 6 layers, attention heads = 8, MLP hidden dimension = 2048, dropout rate = 0.1, and embedding dropout = 0.1. The model leveraged self-attention mechanisms to establish long-range dependencies across spatially encoded 3D patches, enabling hierarchical representation learning of tumor heterogeneity.

Supplementary Table 1:Radiomics Model Evaluation Results

Model Name	Accuracy	AUC	95% CI	Sensitivity	Specificity	PPV	NPV	Precision	Recall	F1	Threshold
AdaBoost train	0.833	0.926	0.789 - 0.878	0.877	0.793	0.797	0.874	0.797	0.877	0.835	0.5
AdaBoost val	0.722	0.781	0.640 - 0.804	0.618	0.817	0.756	0.700	0.756	0.618	0.680	0.5
AdaBoost test	0.667	0.715	0.595 - 0.739	0.633	0.698	0.658	0.674	0.658	0.633	0.645	0.5
GradientBoosting train	0.889	0.969	0.851 - 0.926	0.831	0.943	0.931	0.857	0.931	0.831	0.878	0.5
GradientBoosting val	0.757	0.767	0.678 - 0.835	0.509	0.983	0.966	0.686	0.966	0.509	0.667	0.5
GradientBoosting test	0.703	0.771	0.633 - 0.773	0.570	0.826	0.750	0.676	0.750	0.570	0.647	0.5
LR train	0.811	0.879	0.764 - 0.858	0.777	0.843	0.821	0.803	0.821	0.777	0.798	0.5
LR val	0.722	0.831	0.640 -	0.600	0.833	0.767	0.694	0.767	0.600	0.673	0.5

			0.804								
LR test	0.642	0.746	0.569 - 0.716	0.595	0.686	0.635	0.648	0.635	0.595	0.614	0.5
MLP train	0.822	0.911	0.777 - 0.868	0.800	0.843	0.825	0.819	0.825	0.800	0.812	0.5
MLP val	0.722	0.819	0.640 - 0.804	0.582	0.850	0.780	0.689	0.780	0.582	0.667	0.5
MLP test	0.703	0.766	0.633 - 0.773	0.633	0.767	0.714	0.695	0.714	0.633	0.671	0.5
Naivebayes train	0.719	0.781	0.665 - 0.772	0.685	0.750	0.718	0.719	0.718	0.685	0.701	0.5
Naivebayes val	0.704	0.748	0.621 - 0.788	0.564	0.833	0.756	0.676	0.756	0.564	0.646	0.5
Naivebayes test	0.679	0.716	0.608 - 0.750	0.570	0.779	0.703	0.663	0.703	0.570	0.629	0.5

Supplementary Table 2: The difference in the area under the ROC curve for paired samples in the test set（Radiomics）

Test results are correct.	z	P^a	AUC Difference	Standard error value[b]	95% confidence interval	
					Lower limit	Upper limit
AdaBoost-Gradient Boosting	-1.532	0.126	-0.056	0.274	-0.128	0.016
AdaBoost-LR	-0.815	0.415	-0.031	0.277	-0.105	0.043
AdaBoost-MLP	-1.251	0.211	-0.051	0.275	-0.131	0.029
AdaBoost-Naive Bayes	-0.023	0.981	-0.001	0.282	-0.081	0.079
Gradient Boosting-LR	0.698	0.485	0.025	0.270	-0.045	0.096
Gradient Boosting-MLP	0.158	0.875	0.005	0.267	-0.058	0.068
Gradient Boosting-Naive Bayes	1.615	0.106	0.055	0.274	-0.012	0.122
LR-MLP	-1.019	0.308	-0.020	0.269	-0.059	0.018
LR-Naive Bayes	0.946	0.344	0.030	0.277	-0.032	0.092
MLP-Naive Bayes	2.039	0.041	0.050	0.274	0.002	0.098

a. Null Hypothesis: True regional difference = 0

b. According to non-parametric assumptions

Supplementary Table 3:Habitat Model Evaluation Results

Model Name	Accuracy	AUC	95% CI	Sensitivity	Specificity	PPV	NPV	Precision	Recall	F1	Threshold
AdaBoost train	0.793	0.907	0.744 - 0.841	0.692	0.886	0.849	0.756	0.849	0.692	0.763	0.5
AdaBoost val	0.739	0.784	0.659 - 0.819	0.600	0.867	0.805	0.703	0.805	0.600	0.688	0.5
AdaBoost test	0.709	0.741	0.640 - 0.778	0.608	0.802	0.738	0.690	0.738	0.608	0.667	0.5
GradientBoosting train	0.911	0.987	0.877 - 0.945	0.838	0.979	0.973	0.867	0.973	0.838	0.901	0.5
GradientBoosting val	0.739	0.849	0.659 - 0.819	0.600	0.867	0.805	0.703	0.805	0.600	0.688	0.5
GradientBoosting test	0.721	0.820	0.653 - 0.790	0.595	0.837	0.770	0.692	0.770	0.595	0.671	0.5
LR train	0.937	0.981	0.908 - 0.966	0.915	0.957	0.952	0.924	0.952	0.915	0.933	0.5
LR val	0.765	0.846	0.688 -	0.691	0.833	0.792	0.746	0.792	0.691	0.738	0.5

			0.843								
LR test	0.782	0.805	0.719 - 0.845	0.722	0.837	0.803	0.766	0.803	0.722	0.760	0.5
MLP train	0.948	0.993	0.922 - 0.975	0.923	0.971	0.968	0.932	0.968	0.923	0.945	0.5
MLP val	0.809	0.881	0.737 - 0.881	0.709	0.900	0.867	0.771	0.867	0.709	0.780	0.5
MLP test	0.752	0.817	0.686 - 0.817	0.696	0.802	0.764	0.742	0.764	0.696	0.728	0.5
NaiveBayes train	0.741	0.846	0.688 - 0.793	0.562	0.907	0.849	0.690	0.849	0.562	0.676	0.5
NaiveBayes val	0.652	0.668	0.565 - 0.739	0.400	0.883	0.759	0.616	0.759	0.400	0.524	0.5
NaiveBayes test	0.667	0.747	0.595 - 0.739	0.430	0.884	0.773	0.628	0.773	0.430	0.553	0.5

Supplementary Table 4: The difference in the area under the ROC curve for paired samples in the test set（Habitat）

Test results are correct.	z	P[a]	AUC Difference	Standard error value[b]	95% confidence interval	
					Lower limit	Upper limit
AdaBoost-Gradient Boosting	-2.474	0.013	-0.080	0.266	-0.143	-0.017
AdaBoost-LR	-1.547	0.122	-0.064	0.272	-0.145	0.017
AdaBoost-MLP	-2.053	0.040	-0.076	0.269	-0.149	-0.003
AdaBoost-Naive Bayes	-0.128	0.898	-0.006	0.280	-0.099	0.087
Gradient Boosting-LR	0.410	0.682	0.016	0.259	-0.059	0.091
Gradient Boosting-MLP	0.108	0.914	0.004	0.256	-0.062	0.069
Gradient Boosting-Naive Bayes	1.715	0.086	0.074	0.267	-0.010	0.158
LR-MLP	-0.526	0.599	-0.012	0.260	-0.057	0.033
LR-Naive Bayes	1.537	0.124	0.058	0.271	-0.016	0.132
MLP-Naive Bayes	2.010	0.044	0.070	0.268	0.002	0.138

a. Null Hypothesis: True regional difference = 0

b. According to non-parametric assumptions
